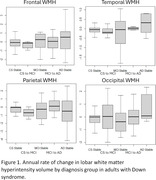# Longitudinal changes in neuroimaging markers of small vessel disease: Implications for clinical trials

**DOI:** 10.1002/alz.092029

**Published:** 2025-01-09

**Authors:** Patrick J. Lao, Natalie C. Edwards, Lisi Flores‐Aguilar, Batool Rizvi, Anna C. Smith, Dana Tudorascu, H. Diana Rosas, Michael A. Yassa, Benjamin L Handen, Bradley T. Christian, Jose Gutierrez, Donna M. Wilcock, Elizabeth Head, Adam M. Brickman

**Affiliations:** ^1^ Columbia University Irving Medical Center, New York, NY USA; ^2^ University of California, Irvine, Irvine, CA USA; ^3^ University of Pittsburgh, Pittsburgh, PA USA; ^4^ Massachusetts General Hospital, Harvard Medical School, Boston, MA USA; ^5^ University of Wisconsin‐Madison, Madison, WI USA; ^6^ Indiana University School of Medicine, Stark Neurosciences Research Institute, Department of Neurology, Indianapolis, IN USA; ^7^ Department of Neurology, Columbia University, New York, NY USA

## Abstract

**Background:**

Adults with Down syndrome (DS) overproduce amyloid precursor protein, develop amyloid plaques at an early age, and are diagnosed with Alzheimer’s disease (AD) dementia at a high frequency. There is emerging evidence that cerebrovascular disease is elevated across the AD continuum in older adults with DS, independent of age and vascular risk, around the same time as amyloid and tau, but the regional rates of accumulation within individuals are unknown.

**Method:**

Adults with DS from the multisite Alzheimer’s Biomarker Consortium‐Down Syndrome study (ABC‐DS; n=78; age=50±6; 40% women) have two timepoints of T2 FLAIR MRI (1.2±0.6 years apart) quantified as white matter hyperintensity volume (WMH), which represents ischemic small vessel disease. Participants underwent consensus diagnosis at both timepoints (59% Cognitively‐Stable at both timepoints, 9% Cognitively‐Stable to MCI‐DS, 8% MCI‐DS at both timepoints, 14% MCI‐DS to AD, 10% AD at both timepoints). The annual rate of change in frontal, temporal, parietal, and occipital WMH volume was assessed, adjusting for baseline WMH volume.

**Result:**

The annual rate of change in frontal WMH was not significantly different by diagnosis. The annual rate of change in temporal (0.7 [0.4, 1.1], p<0.001) and in occipital WMH (1.6 [0.7, 2.5], p=0.0008) was faster in the group that remained AD at both timepoints compared to the group that remained Cognitively‐Stable at both timepoints. The annual rate of change in parietal WMH was greater in the group that progressed from MCI‐DS to AD (0.6 [0.1, 1.0], p=0.02) and in the group that remained AD at both timepoints (1.1 [0.6, 1.7], p=0.0002) compared to the group that remained Cognitively‐Stable at both timepoints.

**Conclusion:**

In adults with DS, parietal WMH accumulates fastest in those that progress to or have a diagnosis of AD, while temporal and occipital WMH accumulate fastest in those with a diagnosis of AD. Posteriorly distributed WMH may have specificity for AD progression in adults with DS with implications for anti‐amyloid therapeutics that have cerebrovascular side effects.